# Trajectory-Aware Offloading Decision in UAV-Aided Edge Computing: A Comprehensive Survey

**DOI:** 10.3390/s24061837

**Published:** 2024-03-13

**Authors:** Tanmay Baidya, Ahmadun Nabi, Sangman Moh

**Affiliations:** Department of Computer Engineering, Chosun University, 309 Pilmun-daero, Dong-gu, Gwangju 61452, Republic of Korea

**Keywords:** unmanned aerial vehicle, mobile edge computing, UAV-aided edge computing, task offloading, offloading decision, trajectory planning

## Abstract

Recently, the integration of unmanned aerial vehicles (UAVs) with edge computing has emerged as a promising paradigm for providing computational support for Internet of Things (IoT) applications in remote, disaster-stricken, and maritime areas. In UAV-aided edge computing, the offloading decision plays a central role in optimizing the overall system performance. However, the trajectory directly affects the offloading decision. In general, IoT devices use ground offload computation-intensive tasks on UAV-aided edge servers. The UAVs plan their trajectories based on the task generation rate. Therefore, researchers are attempting to optimize the offloading decision along with the trajectory, and numerous studies are ongoing to determine the impact of the trajectory on offloading decisions. In this survey, we review existing trajectory-aware offloading decision techniques by focusing on design concepts, operational features, and outstanding characteristics. Moreover, they are compared in terms of design principles and operational characteristics. Open issues and research challenges are discussed, along with future directions.

## 1. Introduction

Internet of Things (IoT) devices and their applications with diverse quality-of-service (QoS) requirements have led to an anomalous demand for computation-intensive and latency-sensitive tasks, including real-time online gaming, image or video processing, autonomous vehicles, augmented reality (AR), and virtual reality (VR) [1]. However, IoT users often face limitations owing to resource constraints, including limited computational resources and energy, making it challenging to execute related applications effectively [2]. Addressing the incompatibility between resource-hungry mobile applications and resource-constrained users has become a crucial challenge for IoT systems [3].

In the dynamic landscape of technology, the development of the Internet and IoT devices has reshaped the manner in which we interact with data and services. The conventional centralized processing model of cloud computing encounters challenges in handling the evolving demands of users, particularly with the sudden increase in IoT devices and the expanding Internet ecosystem [4]. Coupled with the extensive adoption of IoT devices, the Internet has paved the path to an era in which data are generated at revolutionary scales and speeds. From wearable devices and smart homes to connected vehicles and industrial sensors, the diversity and volume of data produced necessitates a paradigm shift in computing architectures [5]. However, as the Internet and IoT ecosystems mature, new challenges have emerged. The limitations of the cloud, including high latency, bandwidth constraints, and elevated energy consumption, have become apparent, particularly in scenarios that demand real-time responsiveness [6]. In the context of mobile communication, particularly with the advent of 5G, the demand for high-quality wireless services grows exponentially [2]. New business scenarios, such as autonomous driving, AR, VR, and diverse applications in smart homes, cities, smart grids, agriculture, and environmental monitoring, pose higher requirements for critical technical indicators, such as time delay, energy efficiency, and reliability [7].

The revolutionary concept of mobile edge computing (MEC) has emerged to bridge the gap between the limitations of cloud computing and the exponential needs of users in the 5G era [8]. MEC brings computational and storage resources to the edge of a mobile network, revolutionizing the processing of demanding applications [9]. Unlike the centralized processing model of cloud computing, MEC positions resources closer to users and devices, facilitating low-latency, high-performance computing directly at the network edge [9]. MEC servers positioned at the edge of wireless networks provide robust computing services with minimal transmission and execution delays. The edge servers can face resource scarcity issues while executing too many tasks, leading to a degraded QoS for the assigned tasks. To address this challenge, various enabling technologies assist edge computing. For example, with the proliferation of Internet of Vehicles (IoV), vehicles can reduce the workload of edge servers. As autonomous vehicles are equipped with substantial computing resources, they can significantly enhance the task processing performance in MEC scenarios. This introduces a new computing paradigm known as vehicular edge computing (VEC), where both parked and moving vehicles contribute to edge computing capabilities [10].

A novel paradigm, the unmanned aerial vehicle (UAV)-aided MEC has recently garnered significant attention from both academia and industry. UAVs provide on-demand deployment, cost-effectiveness, controllable maneuverability, high cruising altitudes, and line-of-sight (LoS) connectivity, making them ideal platforms for edge computing [11]. This approach complements terrestrial networks, particularly when the ground base station (GBS) servers are overloaded or unavailable. Owing to their high mobility and exceptional maneuverability, UAVs have been employed as MEC platforms to extend communication coverage and upgrade deployment efficiency [12]. Thus, UAV-aided MEC provides intensive flexibility and can convey better support for on-demand computing services than conventional MEC that relies on GBSs [13].

**Figure 1:**
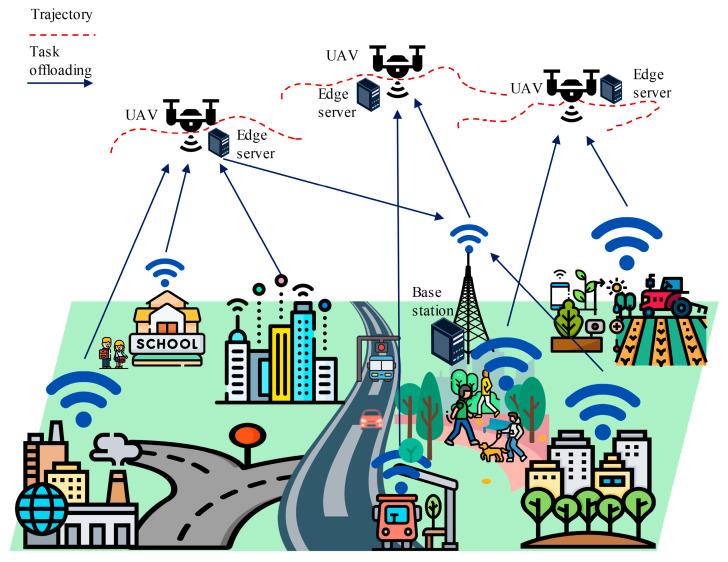
Trajectory-aware offloading decision in UAV-aided edge computing.

**Figure 2:**
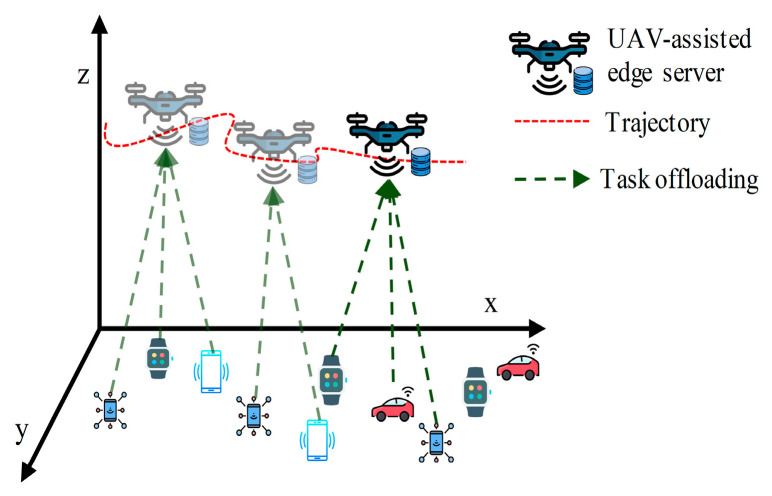
Trajectory-aware offloading decision for single-UAV-aided MEC.

**Figure 3:**
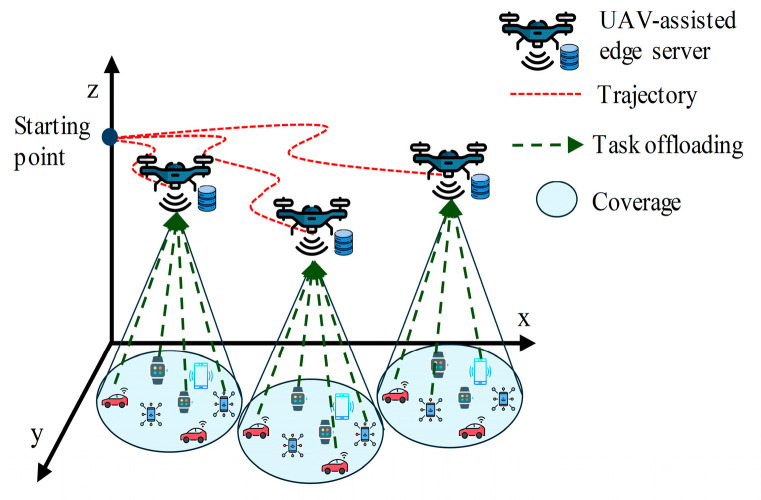
Trajectory-aware offloading decision for multi-UAV-aided MEC.

In the landscape of UAV-aided MEC, the exploration of offloading decisions and trajectory planning is at the forefront of research. Offloading refers to the process of transferring computational tasks to edge servers from a resource-constrained device [14]. When a device faces a task beyond its computation capacity, it can offload the task to an MEC server. Then, the server processes the task and sends the result back to the device. In this context, IoT devices can offload their tasks to the UAV for processing. The UAV then processes the tasks locally or further offloads to the GBS or cloud if a greater processing power is required [15]. Both the IoT devices and UAVs need to take the decision of either computing a task locally or offloading it to the higher layer. This decision is crucial for enhancing the performance and efficiency of the network by maximizing device capabilities, balancing load, and reducing burdens [16]. Appropriate offloading decisions also aid in resource management and cost optimization in space–air–ground-integrated networks by matching resources to the task, reducing network traffic, and enabling real-time application by reducing latency [17]. On the other hand, trajectory planning focuses on determining optimal flight paths for UAVs to effectively serve IoT devices. However, the trajectory planning influences offloading decisions significantly. The distance between a UAV and an IoT device directly impacts latency [18]. When a UAV is far from an IoT device, the device may decide to process the task locally instead of offloading it to the UAV [19]. Therefore, trajectory awareness is essential to enhance the performance and efficiency in offloading decisions. Conventionally, these two features have been independently investigated in the literature, with a focus on either trajectory planning or offloading decisions. However, a recent trend has emerged in which researchers have realized the mutual reliance of these features and are investigating their joint optimization [20]. Offloading decisions are vital for optimizing the overall system performance in UAV-aided MEC, and the trajectory or path of the UAVs significantly influences this decision [16]. Figure 1 depicts an overall view of the trajectory-aware offloading decision process for UAV-aided MEC. A trajectory-aware offloading decision for UAV-aided MEC is delineated for single-UAV and multi-UAV scenarios in Figure 2 and Figure 3, respectively. From Figure 2, we can find that the UAV will follow the path based on the tasks generated by IoT devices. IoT devices offload their tasks to the UAV as the UAV hovers over them along its trajectory. Figure 3 depicts a generalized multi-UAV system. In the case of a multi-UAV system, every UAV has some coverage area according to its computation capability and the remaining energy which is discussed in Section 3 (Key Design Issues). Every UAV hovers over its coverage area to support the IoT devices associated with that area. In particular, IoT devices on the ground offload computation-intensive tasks to UAV-aided MEC servers, and UAV trajectory planning is intricately linked to the location of the IoT devices where the task generation rate is higher [21]. Therefore, in this paper, we aim to comprehensively survey the research articles focusing on the joint optimization of offloading decisions and trajectory planning. Therefore, ongoing studies have emphasized the importance of simultaneously optimizing offloading decisions and trajectories, highlighting the profound impact of trajectory-aware offloading decisions on the overall performance and efficiency of UAV-aided MEC systems.

### 1.1. Related Surveys

Several surveys have been conducted either on computational offloading or trajectory planning in UAV-aided edge computing. In this survey, we will review research articles that concentrate on optimizing both offloading decisions and trajectory planning simultaneously in UAV-MEC systems. Table 1 presents a comparison of existing surveys. However, to the best of our knowledge, trajectory-aware offloading decisions in UAV-aided MEC have not been thoroughly reviewed. The authors of [22] reviewed computational offloading techniques in MEC networks, including offloading objectives and approaches.

In particular, they discussed various key problems regarding offloading objectives, such as the minimization of energy consumption and delay and the maximization of revenue and system utility. In [23], the authors conducted a systematic literature review on the offloading problem in MEC that has been studied in different areas, such as IoT, vehicular edge computing (VEC), radio access networks (RANs), and 5G. In [24], the authors reviewed algorithms used to make offloading decisions in a UAV-enabled MEC. They primarily focused on offloading types and associated algorithms. In [25], the authors focused on the energy efficiency of UAV-enabled MEC. They covered offloading decisions and resource-allocation techniques in their surveys. The authors in [26] discussed privacy issues in offloading and classified various privacy-preserving offloading techniques. Reference [27] discussed the path planning techniques for UAVs. The purpose of UAVs is not restricted to performing edge computing. They cover a wide range of UAV applications in which the trajectory of the UAVs has a significant influence. In addition, they covered the collision avoidance, energy efficiency, and cost-effectiveness of UAV trajectory planning.

Machine learning (ML)- or reinforcement learning (RL)-based offloading techniques and trajectory planning have become popular, owing to their adaptability to dynamic environments. Recently, considerable research has been conducted on ML- and RL-based offloading techniques. The authors of [28] reviewed the ML-based offloading techniques in an MEC environment. According to their taxonomy, these techniques are classified as supervised, unsupervised, or reinforcement-learning-based approaches. Nguyen et al. [29] summarized the RL-based offloading techniques for aerial computing. In [1], the authors systematically reviewed RL techniques used for computation offloading in the overall edge computing. These studies primarily focused on terrestrial edge servers. In [30], the authors reviewed the intelligent offloading techniques used in edge computing. They covered RL- and machine learning-based offloading techniques. The authors of [31] reviewed RL techniques for autonomous UAV navigation. In [32], the authors surveyed and categorized various artificial intelligence (AI) approaches for autonomous UAV navigation. The authors of [33] also surveyed different AI techniques for path planning problems of UAV swarms.

Joint trajectory planning and offloading decisions in UAVs are the most prominent factors that have been studied in recent studies. In [34], the authors provided a comprehensive overview of aerial edge computing (AEC). They considered UAVs as edge service providers. They partially discussed the offloading decisions of the UAVs acting as edge service providers. The authors of [35] analyzed how edge computing and AI influence various technical aspects of UAVs, including power management, formation control, autonomous navigation, privacy and security, and communication. In [36], the authors provided an overview of an aerial MEC. The main focus of this study was the optimization of several challenges, such as resource management, UAV trajectory, and the computation offloading of aerial MEC. They provided a brief overview of aerial MEC.

**Table 1:** Summary of existing surveys on trajectory-aware offloading decision in UAV-aided MEC.

Ref.	Year	UAV-Aided MEC	Offloading Decision	Trajectory Awareness	Focus
[30]	2019	–	●	–	AI frameworks for intelligent offloading techniques in multi-access edge computing (MEC)
[28]	2020	–	●	–	ML-based offloading techniques in MEC environment
[27]	2020	–	–	●	Path planning techniques of UAVs for collision avoidance and energy efficiency.
[25]	2021	●	●	–	Offloading decision and resource allocation approaches to reduce the overall energy consumption in UAV-enabled MEC
[22]	2022	–	●	–	Computation offloading methods in MEC networks, including offloading objectives, approaches, and applications
[31]	2022	–	–	●	RL-based algorithm’s characteristics, abilities, and applications for autonomous UAV navigation
[32]	2022	–	–	●	Categorized various AI approaches for autonomous navigation of the UAV
[33]	2022	–	–	●	AI techniques for path planning problems of UAV swarms
[24]	2022	●	●	–	Offloading techniques and associated algorithms in UAV-enabled MEC
[29]	2022	●	●	–	RL-based offloading techniques in aerial computing
[35]	2022	●	○	●	Showed how edge computing and AI influence various technical aspects of UAVs, including power management, formation control, autonomous navigation, privacy and security, and communication
[36]	2022	●	○	○	Optimization of resource management, trajectory of UAVs, computation offloading of aerial MEC
[23]	2023	–	●	–	Offloading problem in MEC in different applications
[34]	2023	●	○	–	Overviewed the architecture and performance metrics of AEC and partially covered offloading techniques in aerial platforms
[1]	2023	–	●	–	RL-based techniques used for computation offloading in edge computing
Our survey	2024	●	●	●	A brief introduction to UAV-aided MEC systems, highlighting the dependency of trajectory planning and offloading decisions as well as an investigation on recent algorithms and approaches for joint optimization of trajectory planning and offloading decisions, along with a comprehensive comparison.

Note: ● fully covered; ○ partially covered; – not mentioned.

### 1.2. Contribution and Organization

This study conducted an extensive survey on trajectory-aware offloading decisions in UAV-aided edge computing, organizing the research areas for the joint optimization of computation offloading and trajectory design. The main contributions of our survey are as follows:

- We provide a brief overview of the background information on UAV-aided MEC systems, including offloading decisions and UAV trajectory planning. This overview highlights the impact of trajectory planning on offloading decisions within UAV-MEC systems so that general readers can grasp the topic effectively.
- We study the practical application scenarios of UAV-MEC systems. In addition, we discuss key design issues involved in optimizing jointly offloading decisions and trajectory planning.
- A detailed exploration of the recent algorithms and approaches employed in trajectory-aware offloading decisions is provided, shedding light on their methodologies and innovations.
- The algorithms and approaches are rigorously compared to provide readers with valuable insights into their relative strengths and weaknesses.
- The survey critically discusses future research directions and open issues in the field, encouraging further exploration and innovation.

This survey is organized as follows. In Section 2, we describe the background knowledge required to understand the survey. Section 3 discusses the design problems in UAV-aided MEC. In Section 4, we discuss various trajectory-aware offloading decision techniques in terms of their design concepts, operational features, and outstanding characteristics. The reviewed techniques are compared in terms of design principles and operational characteristics in Section 5. Critical issues and challenges are discussed in Section 6 along with possible future directions. Finally, Section 7 concludes the paper.

## 2. Background Study

### 2.1. Edge Computing

In today’s hyper-connected world, data are consumed and generated at a revolutionary rate, driven by the advent of 5G communication networks and the proliferation of IoT devices. Traditional centralized data centers, commonly known as cloud computing, face limitations in handling this massive stream of data, resulting in latency issues, network congestion, and increased energy consumption [37]. In response to these challenges, edge computing has emerged as a transformative paradigm, shifting computing resources closer to the user end of a network where data are either consumed or generated [38].

Edge computing is a distributed computing paradigm that brings computation and storage resources closer to the user end of a network to fulfill the high-performance requirements of users [39]. Edge computing enables users to provide services (communication, computation, and storage) by enabling computations near their physical locations. As the computing services are very close to the users in edge computing, it provides a better QoS to the users because it reduces the distance between the servers and users, which can reduce latency, and for distributed architecture, it can save bandwidth [38].

With the dynamic increase in mobile devices such as smartphones, tablets, autonomous vehicles, AR, and VR, the demand for high-quality wireless services has been growing dramatically. The development of 5G communication in recent years has pushed mobile communication to its future stages. The concept of MEC using edge computing in mobile communications has shown an enormous trend for scholars and developers in both industry and academia. Mobile edge computing has changed our perspective as it plays a pivotal role in every sector of our life, such as smart cities, environment monitoring, smart vehicles, smart agriculture, smart grids, smart industries, and disaster management [40]. Another advantage of MEC is that it can cover any remote area, which is not possible with conventional terrestrial network services.

### 2.2. UAV-Aided Edge Computing

UAVs, commonly known as drones, are equipped with the necessary hardware and software to communicate and compute data and can act as mobile computing platforms. This concept converges UAV and edge computing, creating a new paradigm: UAV-aided edge computing, which can communicate and execute computational tasks at the edge of the network [41]. Since UAVs are autonomous or can be controlled remotely, they are easy to deploy in MEC. There are other advantages to using UAV in edge computing.

- Mobility and versatility: Owing to their high mobility, UAVs can provide on-demand computing power on an emergency basis when traditional infrastructure is absent or limited [42]. As they are equipped with edge computing capabilities, they can collect and process data on-the-fly in real time, which is the most distinctive advantage of UAV-aided edge computing because this characteristic is indispensable in emergency scenarios such as disaster management, surveillance, and environment monitoring, where there is a high demand for instant decision making [43].
- Aerial coverage: As UAVs can operate while flying in the sky, they can easily navigate obstacles and reach challenging areas that traditional terrestrial networks cannot reach. This ability is crucial in certain applications, such as search and rescue operations, environmental monitoring, surveillance, and military operations [44].
- Direct line-of-sight (LoS) communication: UAVs establish direct LoS communication links with ground users, other UAVs or edge devices. This can reduce latency and path loss [45].
- Flexibility: Different UAV models and payload options can be created based on the requirements of specific applications [46].

By their own nature, UAV-aided edge computing institutes have a level of dynamism and adaptability to conventional edge computing paradigms. However, a question arises regarding the number of UAVs considered in edge computing. In some research papers, the authors considered single-UAV-based architectures, whereas others considered multi-UAV-based architectures.

1. Single UAV: In single-UAV-based architectures, only one UAV performs mobile-edge computing. It communicates, collects, processes, and transmits data to all the users. All the responsibilities of MEC rely entirely on that central UAV, although it has both advantages and disadvantages.

*Advantages*: As only one UAV is used in a single-UAV-based architecture, it is extremely easy to deploy and reduces the complexity of the system. Lower infrastructure and maintenance costs make it ideal for budget-constrained projects. 

*Disadvantages*: Limited processing power and area coverage are the main disadvantages of a single-UAV-based architecture. Additionally, a single UAV failure can cause the entire system to crash.

2. Multi-UAV: Collaborative multiple UAVs are involved in a multi-UAV-based architecture, in which each UAV performs its own work while communicating with others to achieve any specific goal. This approach utilizes the collective capabilities of UAVs and distributes tasks to amplify the coverage, efficiency, and overall system resilience [47].

*Advantages*: As multiple UAVs are collaboratively deployed in multi-UAV-based edge computing scenarios, they expand the coverage area of operation and enhance the processing power exponentially [48]. In this model, the failure of a UAV does not crash the entire infrastructure. 

*Disadvantages*: The system becomes extremely complex because it requires many protocols, such as collision avoidance protocols [49]. Moreover, the cost of this architecture is high.

The role of a UAV in the dynamic domain of UAV-aided edge computing has been extended to multifaceted groups with distinct responsibilities. Different studies have shown the different roles of UAVs; some studies consider UAVs as users, whereas others consider UAVs as relays. However, most researchers consider them edge servers.

1. UAV as an MEC server: In UAV-aided edge computing, UAVs can act as edge servers by being transformed into a mobile computing platform capable of on-the-fly data processing at the edge of the network. When a UAV acts as an edge server, its role involves executing computational tasks locally, reducing latency, and minimizing the data transit time [50]. The UAV becomes a self-contained unit as an edge server that handles real-time data analysis and decision making. This role is particularly important in applications that require rapid responsiveness, such as precision agriculture, environment monitoring, and autonomous navigation [35].
2. UAV as a user: The UAV becomes the recipient of the processed data in the role of a user, leveraging the insights generated by edge computing capabilities. This role is crucial in applications where the main objective of the UAV is to receive processed information for further use. For example, UAVs conduct reconnaissance and relay the processed information to a central command station, or they are used in surveillance operations, where real-time insights are vital for informed decision making [51].
3. UAV as a relay: The UAV plays a relay role, acting as a communication link between users and edge servers, facilitating seamless data exchanges. As relays, UAVs have become a pivotal element in creating interconnected networks, ensuring robust communication and coordination, either relaying information between ground devices and the central server or connecting multiple UAVs to form a collaborative fleet. This role enhances the coverage and scalability of UAV-aided edge computing [51]. This is particularly beneficial for applications such as large-scale environmental monitoring, disaster management, or infrastructure inspection.

### 2.3. Offloading Decision

During the proliferation of UAV-aided edge computing, the concept of offloading decisions has played a vital role in shaping the performance and efficiency of UAVs [24]. Offloading refers to the process of transferring a computational task to an edge server (i.e., a UAV) from a resource-constrained device (i.e., an IoT device). When an IoT device faces a task beyond its computation capacity, it can offload the task to a UAV. Then, the UAV processes the task and sends the result back to the device. The UAV can process the tasks locally or further offload to the GBS or cloud if a greater processing power is required [5]. Both the IoT devices and UAVs need to take the decision of either computing a task locally or offloading it to the higher layer. This decision is crucial for enhancing the performance and efficiency of the network by maximizing device capabilities, balancing load, and reducing burdens. A task with higher complexity requires a greater processing power [52]. Hence, the device with lower processing capability decides to offload the task to a UAV or GBS (which has a greater processing capability than IoT devices) [15]. On the other hand, if a task is latency-sensitive, then the device may decide to process the task locally instead of offloading in order to avoid the latency incurred during the offloading process [15]. This is the art of offloading decisions, finding a precise balance between control and efficiency. It refers to the strategic decision regarding where computational tasks are processed on the UAV itself (locally) or at the terrestrial base station of the network [1]. This decision is crucial for optimizing the utilization of resources, reducing latency, and improving the overall system efficiency [24]. This involves a refinement evaluation of factors such as limited resources, task complexity, and communication constraints. Based on recent research, there are three types of offloading decisions.

1. Binary offloading: In binary offloading, the decision is straightforward and simplified into only two options: either the UAV locally processes the computational task, or it offloads the tasks entirely to the base station or cloud. This approach is well suited for scenarios in which the task can be precisely classified based on its complexity and resource requirements [45]. Binary offloading balances efficiency and simplicity and is a practical choice for certain applications.
2. Partial offloading: This is a more granular approach, in which the computational task is divided into two portions based on task complexity and energy requirements. One portion of this task is computed locally in the UAV, and the other portion is offloaded to the base station or cloud [53]. This approach is crucial when particular components of a task require less computational energy and can be processed on the UAV efficiently, whereas other parts require more computational resources and power available at the base station or cloud.
3. Hybrid offloading: In recent studies, hybrid offloading has been considered, and it is more practical to combine the concepts of both binary and partial offloading to provide a dynamic decision-making framework. With the development of heuristics and machine learning algorithms, it is now possible to dynamically adjust the allocation of tasks with hybrid offloading based on real-time conditions [24]. By optimizing resource allocation, hybrid offloading strikes a balance between system scalability, responsiveness, and energy efficiency.

The offloading decision depends on the choice of the computation platform. Recent studies have considered two types of computational platforms: local on the UAV and at the ground edge server.

1. Locally, on a UAV computation platform: This platform directly involves processing computational tasks on the UAV. Local computation is suitable for tasks that require immediate responses and is time-sensitive and computationally lightweight [54]. If the tasks are computed locally, which minimizes latency, local computation is well suited where real-time decision-making is required, such as navigation adjustments and obstacle avoidance.
2. Ground edge server computation platform: This platform involves offloading tasks to the ground edge servers that have greater processing power than the UAV [34]. This approach is advantageous for computationally intensive tasks that require huge processing power beyond the capabilities of a UAV. Thus, ground edge server computation enhances scalability, optimizes resource utilization, and is ideal for applications that require machine learning or complex data analysis.

In the UAV-aided MEC, tasks can be processed either individually or in batches, depending on various factors such as the task size, task complexity, network conditions, and system design preferences. Time-sensitive tasks may be processed individually, while batch processing involves grouping multiple tasks together and executing them simultaneously or sequentially. In practice, a combination of both approaches may be used to optimize the overall system performance and meet diverse application requirements in UAV-aided MEC scenarios.

### 2.4. Trajectory Planning of UAV-Aided Edge Computing

The trajectory planning of a UAV in UAV-aided edge computing is an emerging topology, and many studies have focused on it because without proper trajectory planning, it is not possible to achieve the main purpose of edge computing [55]. The trajectory is the path followed by the UAV while processing computational tasks. Trajectory planning is a strategic trajectory designed to optimize data processing, acquisition, and communication [55,56]. It plays a crucial role in strategically positioning the UAV to collect relevant information at key points along its flight path. This includes identifying optimal locations for computational offloading, ensuring proximity to edge computing resources and adjusting the trajectory based on the dynamic nature of data generation. By intelligently planning the trajectory, the UAV can enhance the quality and quantity of the data collected, supporting more effective edge computing processes. Proper trajectory planning significantly affects the overall system performance.

- Data acquisition optimization: Trajectory planning plays a vital role in optimizing data acquisition by identifying the optimal location. By intelligently planning the trajectory, a UAV can effectively improve the quality and quantity of the data collected [54].
- Efficient task offloading: Trajectory planning and task offloading are closely linked. The trajectory of the UAV must be aligned with its proper position to facilitate efficient task offloading by reducing latency [57].
- Collision avoidance: This is another crucial factor when planning a UAV trajectory. Many recent studies have focused on collision avoidance algorithms and employed them with aviation laws and regulations to ensure the safe navigation of UAVs [31]. While operating, a UAV is required to avoid obstacles, and in a multi-UAV system, it needs to avoid collisions.
- Dynamic flight and energy efficiency: Proper trajectory planning can efficiently minimize the energy consumed by a UAV by optimizing its flight path. Trajectory planning can minimize the energy expenditure by considering factors such as altitude changes, wind conditions, and energy consumption during computational tasks [58].

In the single-UAV-based model, the UAV follows the trajectory based on the task generation of IoT devices. The IoT devices broadcast task offloading notifications, facilitating the UAV to know where tasks need to offload without aimless search, which can effectively minimize the UAV’s flight distance and task completion latency [59]. IoT devices offload their tasks to the UAV as the UAV hovers over them along its trajectory. In the multi-UAV-based model, a central UAV can control the flight paths of the UAVs [33]. In the event of collisions, incidents, or failures, the situations are managed through robust collision avoidance algorithms, fault detection mechanisms, and dynamic re-routing strategies.

### 2.5. Effects of Trajectory on Offloading Decision

**Figure 4:**
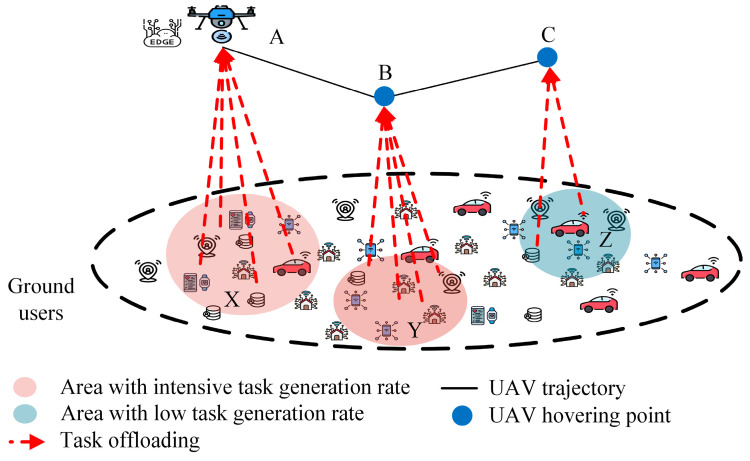
Effects of trajectory design in offloading decision.

In the realm of UAV-aided edge computing, trajectory planning involves not only the flight path of a UAV but also a strategic course that significantly influences the decision-making process of task offloading [16]. In recent years, many studies have focused on joint trajectory design and offloading decisions. Figure 4 illustrates the effects of the trajectory design on offloading decisions.

As edge computing platforms, UAVs should follow a trajectory that covers the maximum support. The trajectory should be planned based on the task generation rate so that the UAV can efficiently collect and process the maximum amount of data. As shown in Figure 2, the UAV is located at position A and processes the tasks generated or consumed by ground users in region X. Upon completing these tasks, the UAV must enter region Y or Z. However, region Y has a higher task generation rate than region Z. Therefore, if the UAV follows the trajectories of A to B and then B to C instead of A to C and then C to B, it will be able to process the maximum amount of data. Offloading decisions are also made based on the trajectory. If the UAV’s trajectory does not cover a device, or the transmission energy for the offloading task becomes very high, then the IoT devices decide to compute the task locally.

The hovering position and time of a UAV have a significant role in offloading decisions. The hovering position should be at the optimal distance from the area where either the task generation rate is higher or the density of IoT devices is higher. The hovering duration should be maximized in these regions to facilitate efficient task offloading. If a UAV stays far from these regions and spends more time elsewhere, resulting higher latency, then the IoT devices will not offload tasks but process them locally. Proper hovering positions and durations efficiently enhance performance while both minimizing latency and maximizing efficiency.

For multi-UAV scenarios, each IoT device has to choose one UAV to offload its task. The IoT devices choose the UAV that can process their tasks with minimum latency. If a UAV has an available processing capacity, the IoT device chooses the UAV that is in the minimum distance. However, if a UAV has no available processing capacity, the IoT device selects the next nearest UAV. Generally, in the multi-UAV system, each UAV has a fixed region to support. In this case, each UAV should be positioned at the center of the designated region to enhance coverage and efficiency.

### 2.6. Application Scenarios of UAV-Aided MEC

The network infrastructure of UAV-aided MEC can be applied across various domains due to its versatility and capability to extend computational resources to remote or dynamic environments. Some practical application scenarios include the following:

1. Disaster management: A UAV-aided MEC network can be deployed in disaster-stricken areas to provide real-time data processing for emergency responders. They can perform tasks such as image analysis for damage assessment and communication relaying to establish connectivity in areas with damaged infrastructures [60]. To cope with disasters like the COVID-19 pandemic, UAV-MEC can be applied to address various challenges. Telemedicine provisions and contact tracking are such challenges where authorities need to process a huge amount of data daily [24]. In such cases, UAVs can be used to deliver medical supplies, measure body temperature, monitor patients from a certain distance, and even detect patients using face recognition [61]. For deployment, UAVs can collect data and then process it locally or offload the data to the MEC server for further processing [62].
2. Search and rescue: UAVs can autonomously navigate through complex environments and perform various tasks, including real-time data processing and communication [63]. UAVs equipped with computing resources can analyze sensor data (such as thermal imaging or LiDAR scans) to detect survivors or threats with high accuracy and speed [64]. Moreover, MEC enables UAVs to offload computation-intensive tasks (such as image or video processing) to nearby edge servers, allowing for the rapid analysis of large datasets and the extraction of actionable insights [65].
3. Environment monitoring: UAVs, equipped with various sensors, can collect data on air quality, water pollution, and biodiversity in remote or risky environments where traditional terrestrial networks cannot be reached [66]. MEC capabilities enable UAVs to process collected data locally for immediate analysis and decision making, without depending on centralized servers. This distributed computing ensures a rapid response to emerging environmental concerns such as wildfires, pollution hotspots, or natural disasters [67]. Moreover, based on environmental feedback and predictive analytics, UAVs can adapt their flight path, which optimizes data collection efficiency and coverage [68]. By offloading data processing tasks to edge servers, we can analyze environmental data in real time, facilitating the early detection of environmental threats [69].
4. Precision agriculture: UAVs equipped with sensors (such as LiDAR scanners, multi-spectral cameras, and thermal imaging devices) can capture high-resolution data on crop health, soil moisture levels, pest infestations, and other vital agronomic parameters [70]. By offloading computational tasks to edge servers, farmers can make timely decisions regarding fertilization, irrigation, and pest control, optimizing crop yield and resource utilization [71].
5. Smart cities: A UAV-aided MEC system is a transformative solution for addressing various urban challenges and enhancing the quality of life for residents. UAVs are equipped with sensors, cameras, and communication devices to collect and process data in real time at the network edge [72]. Thus, UAVs are enabled to analyze diverse urban parameters such as infrastructure integrity, traffic flow, air quality, noise levels, and public safety incidents [73]. The decentralized approach of UAV-MEC systems reduces latency, improves scalability, and enhances resilience to network failures. For example, UAVs can monitor traffic congestion dynamically, identify accidents, and optimize traffic signal timings in real time for enhancing transportation efficiency [74]. Similarly, UAVs equipped with environmental sensors can detect pollution sources, monitor air quality levels, and alert authorities to take proper actions to reduce environmental damage [67]. Moreover, MEC-enabled UAVs can support public safety initiatives by providing aerial surveillance, incident detection, and crowd monitoring capabilities during large-scale events or emergencies [75]. Additionally, UAV-MEC networks can leverage the automation of industry by processing the tasks of industrial IoT devices [76]. Overall, UAV-aided MEC systems contribute to the development of smarter, safer, and more sustainable cities.

## 3. Key Design Issues

Several key issues have been considered in the exploration of trajectory-aware offloading decisions in UAV-aided edge computing. For example, the distributions of ground users play a crucial role in offloading decisions and trajectory planning [77]. Task information, such as task size, task complexity, and latency requirements, directly modifies the offloading decision [45]. For trajectory planning, the height of the UAV from the ground and the consideration of various obstacles are crucial [54]. An energy consumption model is also important for deploying UAV-aided edge computing [78]. In this section, the central design issues are addressed.

### 3.1. Ground User Distribution

In trajectory-aware offloading decisions, a crucial design consideration is the distribution of ground users (i.e., IoT devices, mobile users, and other entities). The geometrical arrangement of these users significantly influences both offloading decisions and trajectory planning [19]. The most common distributions of users considered by researchers include the following:

- Uniform distribution: Uniform distribution indicates an equal probability of users located anywhere in an area [79]. This distribution is crucial for frameworks in which a uniform and balanced coverage of offloading services is expected. When UAV-aided edge computing provides equal resource utilization across the entire coverage area, ensuring a more uniform user experience, the user distribution is considered.

*Advantages*: Uniform distribution confirms the equal probability of the location of users anywhere in the coverage area, ensuring balanced resource utilization across that area. This alleviates hotspot problems and provides uniform and balanced offloading.

*Disadvantages*: It fails to utilize resources efficiently in practical scenarios. This distribution wastes resources in areas where the user density is lower, whereas it cannot fulfill the demand for resources where the density is higher.

- Normal distribution: Ground users are often modeled using normal distribution. Normal distribution reflects a probability density function that forms a bell-shaped curve [80]. This distribution is desirable when the expected user concentrations in certain areas are high, allowing trajectory planning to be sensitive to these areas. It is also vital in scenarios where particular areas experience higher task demands.

*Advantages*: Normal distribution provides a realistic representation of the user density. It allows efficient trajectory planning to serve areas with higher user concentrations and ensures optimized offloading in areas of higher demand. 

*Disadvantages*: It may be impractical in scenarios in which unexpected variations or spikes in user activity occur.

- Random distribution: This introduces unpredictability into the user distribution. The distribution represents real-world scenarios in which the user locations change over time [81]. It enhances the robustness of trajectory planning and offloading decisions by ensuring adaptability to unpredictable and dynamic user patterns.

*Advantages*: A random distribution makes trajectory planning and offloading decisions more robust by simulating real-world scenarios with varying user locations. Adaptability is pivotal for coping with evolving and dynamic user patterns.

*Disadvantages*: The unpredictable nature of random distribution may create challenges for efficient resource allocation and lead to inefficient trajectory planning and the underutilization of offloading capabilities.

### 3.2. Task Information

Another crucial design consideration in trajectory-aware offloading decisions is the characterization and availability of task information. Task information includes parameters such as task size, task complexity, and latency requirements, each of which plays a crucial role in efficient offloading decisions.

- Task size: The size of a task, which represents its computational workload, directly affects offloading decisions. Smaller tasks have relatively low computational requirements and may be well suited for local processing. Therefore, smaller tasks minimize the need for offloading and reduce communication overhead [1]. However, larger tasks may require offloading to the edge or cloud for efficient computation.
- Task complexity: It represents the computational sophistication and intricacy involved in the execution of that task. Additionally, it directly influences the offloading decisions by selecting appropriate computational resources [13]. Simpler tasks can be processed efficiently and locally to avoid unnecessary overhead offloads. However, complex tasks must benefit from the offloading of powerful edges or cloud resources in a timely and efficient manner.
- Latency requirement: The latency requirement of a task is the maximum allowable duration for completing the task. This is crucial for real-time time-sensitive applications. Tasks with rigorous latency constraints may require local processing or offloading to nearby edge servers to satisfy real-time demands [43].

### 3.3. UAV Trajectory and Deployment

In UAV-aided edge computing, the trajectory and deployment of UAVs are crucial design considerations that directly affect the effectiveness and efficiency of MEC. The key elements of this design are as follows:

- Obstacle avoidance: This involves navigating UAVs around physical obstacles, such as trees, buildings, other aircraft, and other structures in the environment [35]. Obstacle avoidance strategies in trajectory planning are extremely crucial for ensuring the integrity and safety of UAV operations [82]. By dynamically and intelligently navigating around obstacles, trajectory planning can optimize the path of the UAV, minimize the consumed energy and risk of collisions, and enhance the overall system reliability.
- Deployment height: This refers to the altitude at which the UAVs operate during their trajectories. The deployment height influences various factors such as energy consumption, communication range, and the UAV’s ability to efficiently offload tasks [83]. Therefore, a proper height consideration is necessary to enhance the overall performance of UAV-aided MEC.

### 3.4. Energy Consumption Model

Another crucial design consideration is the energy consumption model, which includes various energy components essential for optimizing the overall efficiency of UAV-aided MEC networks.

- Transmission energy: This refers to the energy consumed for the communication of data between the UAV and users, clouds, or other UAVs. Modeling and understanding transmission energy are crucial for trajectory planning because they influence the decision to offload tasks or process them locally [84].
- Processing energy: Processing energy refers to the energy required for computing tasks, either locally on the UAV or on the cloud servers. The accurate modeling of processing energy guides the selection of optimal computational resources based on the energy efficiency of each processing option [4].
- Flying energy: This is the energy consumed while the UAV is in motion or flying. Combining flying energy into an energy consumption model is pivotal for trajectory planning because it assists in determining energy-efficient routes and altitudes for UAVs during task offloading [85].
- Hovering energy: This is the energy consumed when a UAV remains stationary at a particular location. It is crucial to consider the hovering energy in trajectory planning as it affects decisions concerning the duration and frequency of a UAV hovering at different locations, aiming for energy-efficient offloading strategies [52].

## 4. Trajectory-Aware Offloading Decision Algorithms

**Figure 5:**
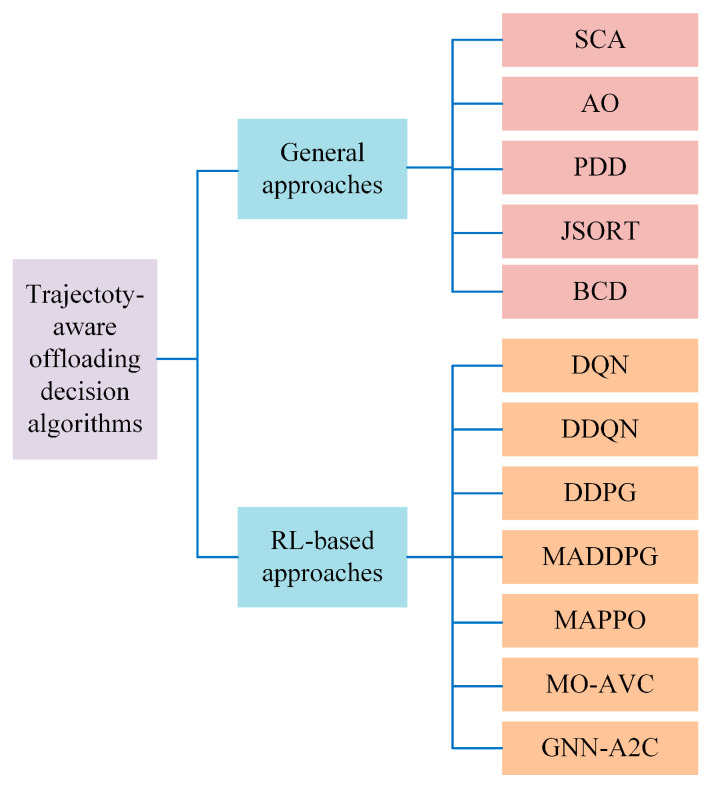
Classification of trajectory-aware offloading decision algorithms: SCA [57], AO [86], PDD [20], JSORT [87], BCD [88], DQN [15], DDQN [89], DDPG [90], MADDPG [91], MAPPO [92], MO-AVC [13], and GNN-A2C [93].

In this section, we compare the different algorithms used for joint trajectory and computational offloading optimization in the existing research. We divided the algorithms into two types: general optimization and RL-based algorithms. Figure 5 illustrates a detailed taxonomy of the optimization algorithms used for trajectory-aware offloading decision making in UAV-aided MEC.

### 4.1. General Approaches

In this subsection, we explore general optimization techniques which are frequently utilized to tackle the complexities of joint trajectory control and offloading decisions in UAV-aided MEC systems. These methods play a crucial role in enhancing system efficiency and performance.

#### 4.1.1. Successive Convex Approximation (SCA)

The SCA is an optimization technique that is particularly used for solving non-convex optimization problems. In the context of trajectory-aware offloading in UAV-aided MEC, the SCA is employed to iteratively solve complex optimization problems, such as resource allocation, trajectory planning, and offloading decision-making processes [94]. In this technique, the non-convex problem is broken down into a series of convex subproblems. The technique begins with an initial approximation and then iteratively refines the solution by solving a series of convex optimization problems. Each iteration aims to upgrade the approximation of the original non-convex problem. This iterative process continues until a satisfactory solution or convergence criterion is achieved. In [57], the authors employed an SCA-based algorithm to reduce the overall energy consumption through a joint optimization approach that considered factors such as CPU frequency, the number of offloaded tasks, transmit power, and UAV trajectory.

*Lessons learned*: The SCA conquers non-convex optimization problems by cleverly replacing them with a series of easier-to-solve convex subproblems. Similar to climbers scaling a rugged mountain, the SCA tackles difficult slopes one manageable step at a time. This flexible approach has proven to be effective in diverse fields; however, it has its trade-offs. On the positive side, the SCA offers convergence guarantees and can be customized to specific problems. However, its performance is sensitive to the initial conditions and may not always reach an absolute peak. Overall, the SCA remains a valuable tool for navigating the unpredictable terrain of non-convex optimization, demonstrating the ability to break down complex challenges into bite-sized pieces. 

#### 4.1.2. Alternative Optimization (AO)

It solves optimization problems by iteratively optimizing a simpler subproblem. In [86], the authors applied an algorithm using a block alternating descent approach to reduce the combined energy consumption of UAV and UDs. This was achieved by jointly optimizing the computational resource allocation and UAV trajectory while adhering to the constraints on the number of computation bits. Moreover, in [16], the authors proposed an algorithm based on the Lagrange duality method to reduce the total weights of three performance metrics: the mean peak age of information (AoI), mean energy consumed by IoT devices, and mean energy consumed by UAVs. The optimization was achieved by jointly adjusting the offloading parameters and trajectories of the UAVs.

*Lessons learned*: The AO conquers complex problems by dividing them into bite-sized subproblems and tackling them one at a time, similar to a team of specialists. This efficient approach is useful in various areas, reaching solutions faster than traditional methods. However, the success of the AO hinges on the structure of the problem and can sometimes become stuck in local optima instead of the global peak. Similar to any good team effort, careful initialization and the selection of the right sub-problems are crucial for the AO to truly shine.

#### 4.1.3. Penalty Dual Decomposition (PDD)

This is based on the idea of dual decomposition, which is a technique for decomposing a problem into a series of smaller and more manageable subproblems. PDD optimizes subproblems independently by introducing penalties to violate the coupling constraints. Higher penalties require stricter compliance. Through iterative updates of the dual variables and penalty parameters, the subproblems gradually coordinate and converge to a solution that satisfies the original set of constraints. PDD efficiently addresses optimization by penalizing violations and iteratively refining the solutions. In [20], the authors used a PDD-based algorithm to reduce the total delay between users within each time slot. It was achieved through the joint optimization of the UAV trajectory, ratio of offloaded tasks, and scheduling variables of the users. This optimization method was conducted while adhering to discrete binary, UAV trajectory, and energy consumption constraints.

*Lessons learned*: PDD offers a powerful and flexible approach for addressing complex optimization problems in scenarios such as joint offloading decisions and trajectory planning in a UAV-aided MEC environment. While it comes with its own challenges, its effectiveness and adaptability make it a valuable tool for researchers and engineers working on demanding optimization tasks.

#### 4.1.4. Joint Stochastic Offloading, Resource Allocation, and Trajectory (JSORT)

A joint optimization algorithm combining stochastic computation offloading, resource allocation, and trajectory scheduling (JSORT) is proposed in [87] to iteratively address the challenge of reducing the average weighted energy consumption of smart mobile devices (SMDs) and UAVs. This optimization is pursued while adhering to the constraints related to resource allocation, computational offloading, and the scheduling of the UAV’s flying trajectory. Recognizing the temporal interdependence of the variables and non-convex nature of the problem, the authors employed a Lyapunov-based method to examine the task queue. The energy consumption minimization problem is then broken down into three more manageable subproblems. To determine the optimal solution, a combination of the alternating direction method of multipliers (ADMMs), interior point method, and CVX solver was deployed.

*Lessons learned*: To tackle the non-convex problem with time-coupled variables, JSORT innovatively employs a Lyapunov-based approach and decomposes the energy minimization task into subproblems. By combining ADMMs, an interior point method, and the CVX solver, JSORT finds the optimal solutions to minimize the average weighted energy consumption of SMDs and UAVs. These lessons emphasize the effectiveness of breaking down complex problems into manageable components and the value of a hybrid approach to optimization in the UAV context. Exploring JSORT’s adaptability to real-world scenarios remains an exciting avenue for future research.

#### 4.1.5. Block Coordinate Descent (BCD)-Based Algorithm

BCD-based algorithms are mostly used in optimization problems where the objective functions can be separable into blocks or variables. Therefore, it is used for large-scale real-world problems where the simultaneous update of all variables is time-consuming and computationally intensive. The main idea of BCD-based algorithms is to optimize and update one block or variable while keeping other variables fixed. It follows the procedure over several iterations until finding the convergence. Researchers need to set the convergence check conditions according to the objective functions. Once the convergence is achieved, the iteration is terminated (which means no update is required); otherwise, the update of each block or variable continues. In [88], the authors implemented a BCD-based algorithm for jointly optimizing communication resources, computation resources, and the trajectory of UAVs to maximize the secure computing capacity. They implemented two blocks to solve the joint optimization problem. In one block, they optimized and updated the time allocation, power allocation, local computing bits, and some auxiliary variables to maximize the secure computing capacity while keeping the fixed trajectory. In the following block, they optimized and updated the trajectory while keeping the other variables fixed.

*Lesson learned*: Most of the real-world UAV-aided trajectory-aware offloading decision problems pose objective functions that can be decomposed into several sub-problems. In these cases, BCD algorithms can be implemented suitably. However, the efficiency of BCD depends heavily on the appropriate choice of variable blocks. Additionally, BCD cannot guarantee global coverage.

*Summary*: The joint optimization of offloading decisions and trajectory planning is generally a non-convex optimization problem which contains both discrete binary variables and the highly coupling constraints [86]. To address such complexity, iterative algorithms such as SCA, AO, and PDD are often employed. These algorithms are preferred over DRL algorithms due to their lower computational complexity, making them suitable for resource-constrained IoT devices [57]. Moreover, these algorithms have higher convergence rates and require less training time and data, making them well suited for real-time deployment scenarios. Additionally, they need lower time to train [20]. Despite their simplicity, these algorithms perform effectively and efficiently, particularly in scenarios with a limited number of IoT devices and UAVs [87]. Moreover, SCA, AO, JSORT, and BCD approaches address compute-intensive tasks while PDD is employed for latency-sensitive applications.

### 4.2. RL-Based Approaches

Recently, RL has gained considerable attention for its capacity to adapt to dynamic environments, rendering it particularly suitable for addressing the evolving nature of UAV-aided MEC systems. In this subsection, we delve into the RL-based optimization strategies utilized in trajectory-aware offloading decisions within UAV-aided MEC networks.

#### 4.2.1. Deep Q-Network (DQN)

The DQN is a reinforcement learning-based algorithm that combines both Q-learning and deep neural networks to optimize the Q-function that represents the expected cumulative rewards for taking a distinct action in a given state. The algorithm is well established for tackling complex non-convex problems, such as the joint optimization problem of task offloading and trajectory planning of a UAV-assisted MEC system [95]. The authors of [15] formulated an optimization problem in a Markov decision process (MDP) environment to solve the non-convex problem associated with both task offloading and trajectory control in a UAV-aided MEC system. The state space includes information related to user devices, task profiles, the distribution model of the network channel, and various parameters of the UAV and its position. The action space involves determining the ratio of offloaded tasks and controlling the upcoming position of the UAV. The reward was defined as the number of tasks completed before expiration minus the associated energy and time consumed by the system. In the proposed end-to-end DQN model, actions were taken to optimize multiple objectives, including computing the latency and overall energy consumed by the system. Specifically, the agent aims to maximize the number of tasks executed before expiration while concurrently minimizing energy and time consumption. Their results suggest that the performance of the proposed model surpasses that of existing approaches.

*Lessons learned*: The DQN showed notable strengths in adapting to dynamic UAV environments and effectively learning the complex spatiotemporal patterns inherent in trajectories. However, challenges have been encountered during its practical implementation. Hyperparameter tuning plays a crucial role in the performance of the algorithm, requiring careful consideration of learning rates, discount factors, and exploration–exploitation balances. The transferability of knowledge from simulated to real-world environments reveals the importance of addressing discrepancies and enhancing generalization capabilities. In terms of advantages, the DQN exhibited advantages in handling nonlinear relationships in trajectory data, contributing to adaptive and efficient offloading decisions. Its ability to learn from experience through iterative updates, particularly using experience replay and target networks, makes it well suited for scenarios with dynamic UAV trajectories. However, achieving the optimal performance often requires accurate hyperparameter tuning, and the real-time computational efficiency of the algorithm increases considerations in resource-constrained UAV environments. 

#### 4.2.2. Double Deep Q-Network (DDQN)

The DDQN is an extension of the DQN and is a reinforcement learning technique designed to optimize decision-making processes in dynamic and complex environments. DDQN enhances traditional Q-learning by mitigating the overestimation problems associated with single-DQN architectures. This is achieved by introducing a separate target network to stabilize the Q-value estimations during learning. A DDQN employs a neural network to approximate the Q-function, enabling it to make informed decisions by iteratively updating its understanding of the environment through interactions. In trajectory-aware offloading decisions for UAV-aided MEC systems, the DDQN is valuable for optimizing resource allocation and trajectory planning by efficiently navigating the trade-offs between exploration and exploitation, ensuring more robust and adaptive decision making in UAV-centric MEC. In [89], the authors employed the DDQN algorithm for resource allocation and trajectory optimization. In this study, the DDQN-based scheme aimed to maximize the average secure computing capacity by jointly optimizing the UAV trajectory, time allocation, and offloading decisions.

*Lessons learned*: The DDQN reinforcement learning framework has demonstrated notable strengths, particularly in mitigating the overestimation problems associated with the traditional DQN. The DDQN stabilizes Q-value estimations by introducing a separate target network, leading to more reliable decision making in dynamic environments. The adaptive learning mechanism of the algorithm enables the effective navigation of the exploration–exploitation trade-off, ensuring robust and efficient trajectory planning. The lessons learned emphasize the significance of the DDQN in optimizing resource allocation and enhancing the security of UAV-aided MEC systems, making it a valuable tool for addressing the complexities of trajectory-aware offloading decisions in dynamic and potentially adversarial environments.

#### 4.2.3. Deep Deterministic Policy Gradients (DDPG)

DDPG is a modern RL algorithm developed to train agents to make decisions in an environment with continuous action spaces. It can learn policies for complex tasks by combining concepts from both value-based and policy-based methods and is particularly well suited for tasks that require the precise control of actions, such as continuous robotic motion control and UAV-aided MEC system joint UAV mobility, user scheduling, and resource allocation optimization. DDPG absorbs an actor–critic architecture; that is, the Q-value action function uses two neural networks. The actor network performs actions by estimating the optimal policy and mapping the states to continuous actions. However, the critic network evaluates these actions by estimating the value function and providing feedback on the quality of the chosen actions. The authors in [90] present an offloading approach derived from DDPG, providing support for a continuous action space. Their approach extended the flexibility of tuning and training a pair of neural networks. The first is an actor network that enhances the Q-value estimated by another network called the critic network. The critic network is responsible for the maximum expected rewards. The proposed solution considers UAV dynamics while minimizing computational costs, which are defined as a normalized function of the processing time delay and energy consumption of the UAV. Moreover, an incorporate mechanism was introduced to handle the uncontrolled dynamics of the UAV, which guides the UAV in learning accumulated errors throughout its trajectory. The methods were applied within a UAV-aided MEC framework. It learns the computation offloading policy effectively and also determines actions such as UAV placement, offloading ratio and the user to be served.

*Lessons learned*: DDPG’s deterministic policy approach has proven beneficial in scenarios where precise and continuous actions, such as UAV trajectory planning, are crucial. The integration of actor–critic networks facilitated the effective learning of optimal policies and value estimations, contributing to adaptive decision making in dynamic UAV environments. Challenges encountered during practical implementation include the need to carefully tune the hyperparameters, particularly those related to actor and critic networks, to achieve the optimal performance. In terms of advantages, DDPG is proficient in providing precise and deterministic decision outputs, which are crucial for trajectory-aware offloading scenarios. Its ability to handle continuous action spaces contributes to its effectiveness in optimizing UAV trajectories and offloading decisions. However, careful attention is required in parameter tuning to harness the full potential of DDPG. As with any reinforcement learning algorithm, the transferability of learned policies to real-world environments requires consideration, emphasizing the importance of enhancing DDPG’s generalization capabilities of DDPG.

#### 4.2.4. Multi-Agent Deep Deterministic Policy Gradients (MADDPGs)

MADDPG is an extension of the DDPG algorithm, designed to address scenarios with multiple interacting agents in a cooperative or competitive setting. It enables multiple agents to learn coordinated policies in a decentralized manner, enabling them to achieve cooperative or competitive goals in a shared environment. This allows multiple agents to learn policies concurrently by considering the effect of each agent’s actions on the overall environment. MADDPG is designed for environments in which multiple agents interact with each other and with the environment simultaneously. Each agent has its own observations of the environment and performs actions independently. Similar to DDPG, MADDPG maintains an actor–critic architecture for each agent. The actor network determines the actions of the agent and the critic network evaluates the chosen actions in the context of the overall environment. The agents are trained jointly, meaning that they share information during training. Each agent’s actor network is trained to maximize its own expected cumulative reward; however, critic networks consider the actions of all agents to evaluate the joint impact. Each agent maintains its own actor and critic networks, learning a policy based on the local observations and actions of other agents. The critic network evaluates the joint actions of all the agents by considering the interactions and interdependencies between them. In [91], the authors proposed the MADDPG algorithm considering power IoT devices operated in an air–ground integrated network (AGIN). The network considers that power IoT devices located on the ground generate computation tasks and offload tasks to UAVs for computation. Given the constrained battery capacity of both power IoT devices and UAVs, the primary aim is to reduce the long-term average power consumption while adhering to queue latency constraints. The proposed MADDPG algorithm achieves this objective through the joint optimization of task offloading, computing resource assignment, and the trajectory planning of UAVs.

*Lessons learned*: MADDPG facilitates effective cooperation by empowering agents to acquire joint policies, thereby ensuring that UAVs consider the broader impacts of their offloading decisions. The practical implementation revealed the significance of carefully tuning the hyperparameters for each agent, emphasizing the decentralized nature of the learning process. MADDPG’s decentralized approach enables UAVs to adapt to dynamic trajectory scenarios while collaboratively optimizing offloading decisions. In terms of advantages, MADDPG is proficient in facilitating collaborative decision making among UAVs, contributing to more efficient trajectory-aware offloading. However, challenges persist in ensuring the stability of multi-agent interactions, and ongoing research is required to refine the generalization capabilities of MADDPG across different collaborative scenarios. 

#### 4.2.5. Multi-Agent Proximal Policy Optimization (MAPPO)

Proximal policy optimization (PPO) is a reinforcement learning algorithm designed to train agents to make decisions in environments with discrete or continuous action spaces. MAPPO is an extension of the PPO algorithm, designed to handle scenarios with multiple interacting agents. In [92], MAPPO was used as a vigorous offloading strategy in an edge computing network. The scenario involves the collaboration of numerous UAVs to serve several user equipment (UE) devices. The authors considered the challenges posed by imperfect channel state information (CSI) among UE and UAVs and the uncertainties associated with the complexity of the tasks. Their main objective was to extend the robustness of the system while simultaneously reducing the total energy consumption. Their goal was achieved by the joint optimization of the trajectory of the UAV, task partitioning, and allocation of communication and computation resources in multi-UAV environments.

*Lessons learned*: MAPPO’s decentralized learning approach allows individual agents to adapt their offloading policies independently, fostering efficient coordination within a multi-agent system. Practical implementation highlights the importance of parameter tuning, particularly for the exploration–exploitation trade-off, to ensure effective learning and adaptation. MAPPO’s emphasis on policy optimization and its inherent ability to handle nonstationary environments contribute to its suitability for trajectory-aware offloading in dynamic UAV scenarios. In terms of advantages, MAPPO provides proficiency in enabling decentralized learning and coordination among UAVs, resulting in more adaptive trajectory-aware offloading decisions. The ability of an algorithm to handle complex dynamic environments is a significant advantage. Challenges include the need for careful parameter tuning and emphasizing the importance of striking a balance between exploration and exploitation. 

#### 4.2.6. Multi-Objective Actor–Variations Critic (MO-AVC)

The MO-AVC algorithm is a promising reinforcement learning (RL) approach for trajectory-aware offloading decisions in UAV-aided MEC. Instead of focusing on a single objective such as minimizing completion time, MO-AVC explicitly considers both performance and risk through a reward function that incorporates penalties for potential offloading failures or resource constraints. It enables the algorithm to learn policies that optimize the trade-off between these competing objectives. MO-AVC utilizes an actor–critic architecture, where the actor network learns a policy that maps the UAV states (e.g., location, battery level, and network conditions) to offloading decisions. In contrast, the critic network estimates the expected value of the chosen action by considering both the potential rewards and penalties associated with its performance and risk consequences. A unique feature of MO-AVC is its ‘variations critic’ module. The module helps the algorithm to assess the potential variability of its policy by estimating the uncertainty associated with the chosen offloading action. It allows MO-AVC to favor decisions that lead to more predictable outcomes, thus minimizing the risk of unexpected performance fluctuations or failures. In [13], the authors investigated an MO-AVC-based solution to address the joint trajectory control and task offloading (JTCTO) problem in a multi-UAV-aided MEC environment. Their primary focus was to reduce the task latency and minimize the energy consumption of the UAV. Their goal was to maximize the number of tasks collected by the UAVs. The simulation outcomes demonstrated the adaptability of the proposed algorithm to new environments, exhibiting a reduced energy consumption, decreased latency, and an increased number of collected tasks compared with existing solutions.

*Lessons learned*: MO-AVC employs an adaptive learning process in which both actor and critic networks continuously update their parameters based on the received rewards and critic evaluations. It enables the algorithm to learn from its experience and refine its policy over time by adapting to changing network conditions and task requirements. Overall, MO-AVC is a promising approach for trajectory-aware offloading in UAV-aided MEC. Its multi-objective formulation, adaptive learning framework, and focus on risk mitigation make it a valuable tool for developers seeking to achieve reliable and efficient task executions in dynamic resource-constrained environments.

#### 4.2.7. Graph Neural Network-Based Actor–Critic (GNN-A2C)

GNN-A2C is a type of robust deep-graph-based reinforcement learning algorithm that combines the strengths of a graph neural network (GNN) and the advantages of actor–critic (A2C) algorithms in solving RL problems in graph-structured environments. A GNN is a type of neural network distinctly designed to process graph data. Graphs comprise nodes (entities or objects) connected by edges (representing the relationships between nodes). A GNN can learn from the relationship between the nodes presented in a graph, which makes it powerful for solving complex problems in graph-structured scenarios. By contrast, A2C is an RL algorithm that combines the elements of both value- and policy-based approaches. Here the ‘actor’ is accountable for decision making and the ‘critic’ is responsible for evaluating these decisions. The A2C trains agents to take action in an environment by estimating the expected future reward of each action from each state. The GNN-A2C algorithm combines the strengths of both the GNN and A2C algorithms, where the GNN is used to extract the features from the graph, and A2C is used to understand a policy for selecting actions from the features. This joint algorithm is highly robust for solving complex problems, such as UAV trajectory control and task offloading. In [93], the authors proposed a GNN-A2C algorithm for offloading tasks and controlling the cruise of a UAV in an aerial edge IoT environment. Their main objective was to maximize the computation tasks offloaded from IoT devices to the UAV.

*Lessons learned*: The GNN-A2C algorithm is a promising solution for trajectory-aware offloading decisions. The adaptability of the algorithm to graph-structured UAV environments effectively leverages the ability of GNNs to discern spatial relationships. The integration of advantageous actor–critic networks enhances decision-making by learning policies from features extracted by the GNN, which is particularly advantageous for optimizing joint cruise control and task offloading. Challenges include hyperparameter tuning nuances and emphasizing the sensitivity of the algorithm to factors such as learning rates. The GNN-A2C algorithm, as explored in [93], underscores its robustness in maximizing computational task offloading from IoT devices to UAVs. These insights contribute to advancing trajectory-aware offloading strategies in UAV-aided edge computing and urge continued explorations to refine hyperparameter tuning for broader applicability in real-world scenarios.

*Summary*: Integrating RL with MEC networks offers enhanced efficacy because RL algorithms work efficiently in dynamic and highly non-linear environments with complex datasets [90]. RL techniques excel in learning complex decision policies from raw data, allowing for more adaptive and context-aware behavior. However, RL algorithms typically demand higher computational costs and require extensive training data, limiting their applicability in resource-constrained devices. Nontheless, these algorithms demonstrate an effective performance, particularly in scenarios with a large number of IoT devices and UAVs [13]. Moreover, DQN and MO-AVC are highlighted for their effectiveness in addressing latency-sensitive tasks, while other RL approaches are emphasized for their capability in handling compute-intensive tasks.

## 5. Comparison

In this section, we compare existing algorithms designed for trajectory-aware offloading decisions in UAV-aided edge computing in terms of design principles (Table 2) and operational characteristics (Table 3).

Table 2 presents a comparison of the algorithms based on the optimization objectives. From Table 2, it is evident that almost all the studies had the same optimization objectives (i.e., to minimize energy and latency). The most common performance metrics are the energy and delay. From Table 2, we can understand that RL-based algorithms are more computationally intensive than traditional algorithms but perform better in uncertain environments that are more likely to be real-world scenarios. Both DQN and DDQN are Q-value-based RL algorithms; however, DQN is widely used to minimize the energy consumption and latency, while DDQN is used to maximize the secure computing capacity. While becoming simple and efficient, DQN suffers from complex state spaces. In contrast, the DDQN can reduce the overestimation bias of the DQN but increases the computational cost. DDPG, MADDPG, and MAPPO are policy-based RL algorithms designed to minimize energy consumption in all studies. DDPG can handle continuous action spaces but requires careful tuning of the hyperparameters. MADDPG and MAPPO are both able to handle multi-agent environments but face training difficulties. MAPPO is more stable than MADDPG. MO-AVC and GNN-A2C are actor–critic-based RL algorithms, both used for minimizing energy and latency and maximizing the computation tasks offloaded. MO-AVC is adaptive in uncertain environments but faces the challenge of tuning the hyperparameters. In contrast, GNN-A2C can handle graph-structured state spaces but introduces a higher computational cost. All other general approaches (i.e., SCA, AO, PDD, and JSORT) aim to minimize the energy or delay. The SCA can exhibit a superior performance for high data volumes, but its implementation is complex. The AO consumes less energy but struggles to handle multi-UAV. PDD ensures a high convergence rate for moving UAVs, and JSORT exhibits higher adaptability in unpredictable environments. 

From Table 3, it can be observed that most of the studies considered a single UAV; however, few studies considered multi-UAV scenarios, which are more practical. For multi-UAV scenarios, RL algorithms are better suited. Almost all the research papers considered a fixed UAV height. User mobility is another crucial operational characteristic that was not considered in older studies. However, recent works consider the user mobility which is more realistic. Hence, user mobility should be considered for future research in UAV-MEC systems. Most studies have used Python-based platforms to set up their environments to deploy RL algorithms. For general approaches, however, MATLAB is used as a simulation tool. From Table 3, we can observe that different task sizes have been considered for different algorithms. In most studies, the UAV altitude was fixed at 10 m or 100 m. In most studies, the UAV speed was fixed. Table 3 indicates that multi-agent or multi-objective-based RL algorithms (i.e., MADDPG, MAPPO, and MO-AVC) are more suitable for handling multiple UAVs.

Finally, the choice of a trajectory-aware offloading decision algorithm is subject to the specific requirements and constraints of the UAV-aided MEC scenarios. While some algorithms focus on energy consumption, others prioritize computational costs, latency, or multi-agent adaptability. Researchers and developers must carefully consider the nuances and trade-offs of each algorithm when selecting and designing solutions.

## 6. Open Issues and Future Research Directions

Although remarkable progress has been achieved in trajectory-aware offloading decisions for UAV-aided edge computing, many open issues and challenges remain. In this section, we discuss the crucial open issues and possible future research directions to provide exciting opportunities for advancement in this dynamic field.

### 6.1. Symbiotic Edge Intelligence

With advancements in technology, the collaborative capability of intelligent systems at the edges of networks has become increasingly pivotal. Symbiotic edge intelligence refers to the collaborative interaction between different intelligent entities located at the edge of a network. Owing to the need for high computing capabilities at the edge, the use of multiple UAVs instead of a single UAV is more practical. Many studies on computation offloading assume that UAVs can directly offload their tasks to a GBS or cloud if more processing is required beyond their capacity. However, UAVs may operate in large regions, post-disaster scenarios, and remote areas where terrestrial networks are not functioning properly. These areas face challenges with impaired communication infrastructures, resulting in the failure to offload tasks directly [96]. In such situations, collaboration among UAVs is necessary so that each UAV offloads tasks to suitable neighboring UAVs. In symbiotic systems, UAVs learn from each other and adapt their behaviors in real time for optimal data collection and processing in a distributed manner. DRL and federated learning are the key technologies in this dynamic field.

*Probable future direction*: Future research should focus on developing vigorous frameworks that accelerate the seamless collaboration between UAVs, edge devices, and computing resources. In symbiotic systems, UAVs need to communicate and share data with each other. Hence, an investigation is needed on communication protocols among UAVs, which decide how, when, and what information will be shared among UAVs for enhancing their collaborative performance [97]. A central UAV can be deployed to find the optimum offloading and routing strategy [96]. By addressing these aspects, researchers can contribute to the establishment of a symbiotic edge intelligence paradigm that optimizes the performance and adaptability of UAV-aided edge computing environments. 

### 6.2. Space–Air–Ground-Integrated Network (SAGIN) with Seamless Collaboration

The concept of SAGIN envisions a holistic network architecture that seamlessly integrates satellite, aerial, and terrestrial communication technologies to enable ubiquitous connectivity and collaboration among diverse platforms [17]. The core idea of SAGIN leverages synergistic interactions between space-based assets (e.g., satellites), aerial vehicles (e.g., UAVs), and ground infrastructures to create a unified communication fabric capable of supporting a wide range of applications and services. However, SAGIN faces some challenges due to its specific characteristics such as heterogeneity, self-organization, and time variability compared to traditional ground or satellite networks [98]. Fortunately, the edge and cloud computing paradigm arises to address these issues by both reducing latency and enhancing reliability [99]. MEC aims to optimize the distribution of computational tasks in a resource-efficient and dynamic manner. With the evolution of IoV, autonomous vehicles including both moving and parked vehicles can assist SAGIN by providing the support of computational resources [10]. This collaborative framework of various network components (that include cloud infrastructure, edge servers, autonomous vehicles, UAVs, and satellites) within a unified network architecture will be the upcoming network.

*Probable future direction*: The key research challenges regarding SAGIN lie in developing efficient communication protocols, routing algorithms, and resource management schemes. Hence, an investigation is needed to address issues such as dynamic network topology management, heterogeneous network integration, spectrum allocation, mobility management, and security and privacy concerns. Moreover, the design of SAGIN-based MEC systems must consider the efficient arrangement of computational tasks and data offloading across multiple network layers and domains to optimize performance, reliability, and energy efficiency. In summary, future research efforts in this area should focus on exploring novel architectures, algorithms, and protocols customized to the specific requirements of SAGIN-based UAV-aided MEC systems.

### 6.3. Blockchain-Powered Security and Privacy

With the increasing data sensitivity in UAV-aided edge computing, traditional security systems are becoming insufficient. The blockchain technology provides a decentralized and tamper-resistant framework that offers revolutionary promise for enhancing the security and privacy of UAV data communication and networking. The UAV-MEC system contains three types of entities: IoT devices, data generated by these devices, and digital evidence from these data interactions [100]. These entities are fully distributed and difficult to protect through conventional centralized authentication and security mechanisms [101]. Blockchain is applied in the UAV-MEC system to protect data during transmissions between IoT devices and edge nodes, and subsequently, it is applied even to cloud servers [100]. When an IoT device joins the UAV-MEC system, the blockchain authorizes it, ensuring data confidentiality and integrity during transmissions to the edge computing server. This technology records all data transactions and decisions, ensuring accountability, transparency, and tamper-free security [102]. However, implementing a blockchain in UAV-aided edge computing raises challenges associated with latency, scalability, and resource constraints.

*Probable future direction*: First, it is necessary to develop efficient and lightweight consensus algorithms customized for the resource constraints of UAVs, thereby guaranteeing that blockchain transactions do not excessively hamper the computational capabilities of these aerial platforms. Furthermore, an investigation is needed to deploy blockchain technology in UAV-MEC networks to address the challenges of latency and scalability in the networks.

### 6.4. User-Centric Design and Human–Drone Interaction

Developing adaptive intuitive interfaces for humans to collaborate in real time with UAVs and optimizing offloading decisions and trajectories based on a shared understanding and evolving priorities is another area of research. Systems that consider real-time input from human operators or users can adjust their UAV trajectories based on user preferences, mission objectives, or unexpected events. This human-centric approach ensures that trajectory-aware offloading decisions align with the intentions and situational awareness of stakeholders. As UAVs become more widespread in various applications, such as agriculture, remote area coverage, disaster management, and military, ensuring inherent and seamless interactions between drones and users is crucial. The challenge exists in developing user-centric design protocols that not only consider the technical features of trajectory-aware offloading but also prioritize human decisions in real time. This also includes designing interfaces, communication protocols, and control frameworks that feed into the diverse preferences of users interacting with UAVs.

*Probable future direction*: Future research should investigate revolutionary methods to enhance the overall user experience and usability of UAV-aided edge computing systems by merging user-centric designs and real-time human–drone interactions. First, exploring inherent and adaptive interfaces that permit users to interact and control UAVs easily while making joint trajectory and offloading decisions is pivotal. This includes an analysis of user behavior, preferences, and cognitive load to create interfaces that are responsive and user-friendly. Second, realizing the ethical and social perspectives of human–drone interaction is crucial. Researchers should focus on developing frameworks that address safety considerations, societal perceptions, and privacy concerns regarding the UAV design. Moreover, incorporating artificial intelligence and machine-learning algorithms to understand user preferences and intentions can contribute to the development of more proactive and user-friendly trajectory-aware offloading systems. As the field progresses, interdisciplinary collaboration between human and computer interaction experts, designers, and engineers is essential to establish a user-centric design approach that maximizes the effectiveness and acceptance of UAV-aided edge computing solutions in various user scenarios.

### 6.5. Virtual Reality Interfaces for Trajectory Visualization

There is a need to develop innovative VR interfaces that enable human operators to visualize and interact with UAV trajectories in real time. As UAVs travel across complex trajectories in various environments, the demand for immersive and intuitive visualization mechanisms has become predominant. The real-time visualization of UAV trajectories can enhance the capability of system administrators to maintain and control UAVs. A challenge exists in developing VR interfaces that provide system administrators, operators, and users with an immersive and geometrically accurate representation of UAV trajectories. To address this issue, technical challenges associated with real-time data streaming, rendering, and user interactions within the VR environment must be addressed while ensuring that the visualization process does not hamper the decision-making process.

*Probable future direction*: Research efforts should focus on exploring techniques for the real-time streaming and rendering of UAV trajectory data in VR environments because this is pivotal for controlling the responsiveness of the interface. However, it is essential to optimize the balance between computational efficiency and visual richness. Investigating user interaction frameworks within VR interfaces is crucial for ensuring that system administrators can seamlessly analyze, navigate, and make decisions based on visualized trajectories. Moreover, analyzing the effects of VR interfaces on decision-making performance and workload is crucial for realizing practical utility. As the field evolves dynamically, multi-disciplinary collaboration among experts in human–computer interaction, VR technology, and UAV-aided edge computing will be crucial for developing interfaces that not only visualize trajectories effectively but also contribute to more informed and efficient trajectory-aware offloading decisions.

### 6.6. Self-Sustaining UAVs

The emergence of UAVs has led to transformative possibilities; however, they depend entirely on conventional energy sources, which limit their autonomy and operational stamina. It is essential to explore the integration of energy-harvesting technologies such as kinetic energy and solar power to create self-sustaining UAVs. The challenge lies in exploring innovative frameworks that enable UAVs to become self-sustaining and capable of harvesting energy from the environment or deploying advanced energy-efficient technologies. This includes exploring technologies such as solar power, energy harvesting, and optimized power management systems to enhance the operational duration and minimize dependency on external power sources. Attaining such self-sustainability will enable UAVs to be deployed in inaccessible or remote areas where a continuous power supply is not possible while enhancing the endurance of UAVs.

*Probable future directions*: Future research should focus on the development of robust technologies to enhance the autonomy and endurance of UAVs. First, it is essential to investigate advanced energy-harvesting methods such as solar panels or kinetic energy recovery systems that are tailored to the unique characteristics of UAVs. This includes optimizing the design and placement of energy-harvesting components to maximize the efficiency without compromising the aerodynamics or functionality of the UAV. Secondly, it is crucial to explore intelligent power management systems that dynamically allocate and prioritize energy usage based on mission objectives and offloading requirements. Moreover, the integration of machine-learning algorithms to predict and adapt to energy availability during trajectory-aware offloading operations can significantly enhance the self-sustainability of UAVs. As the field progresses, interdisciplinary collaboration among experts in energy systems, aerospace engineering, and UAV-aided edge computing will become pivotal for realizing the vision of self-sustaining UAVs capable of prolonged and autonomous operations. Additionally, it is necessary to investigate how UAVs equipped with energy-harvesting capabilities can autonomously adapt their trajectories based on energy availability, thereby leading to more sustainable and efficient offloading decision strategies.

### 6.7. Multi-UAV Collaboration with Enabling Technologies

As UAVs become increasingly integral to various applications, the potential benefits of collaborative operations among multiple UAVs have become apparent. The collaboration of multiple UAVs is a crucial area in UAV-MEC networks, with challenges arising such as load balancing, failover, handover issues, and adaptive trajectories. While many cutting-edge UAV-based models rely on the collaboration of multiple UAVs, there remains a need to explore novel solutions that leverage emerging technologies such as non-orthogonal multiple access (NOMA), reconfigurable intelligence surface (RIS), and integrated sensing and communication (ISAC) for supporting the wireless connectivity of a vast number of IoT devices simultaneously [103]. When these technologies are combined with UAVs for computing offloading, they can offer promising avenues for enhancing collaboration among UAVs.

*Probable future directions*: Researchers should focus on designing algorithms to balance the load of different UAVs in multi-UAV systems. When and how one UAV hands over its tasks to another UAV in the case of a failure should be investigated. Exploring collision avoidance algorithms for efficient and adaptive path planning in crowded airspaces is essential to ensure the safety and effectiveness of collaborative multi-UAV operations. Moreover, the efficient deployment of enabling technologies (i.e., RIS, NOMA, ISAC, etc.) should be studied for unlocking the full potential of multi-UAV collaboration.

## 7. Conclusions

We have conducted a comprehensive and systematic survey of trajectory-aware offloading decision techniques for UAV-aided edge computing. We have introduced background information on UAV-aided MEC systems including offloading decision and trajectory planning and highlighted the effects of trajectory planning on offloading decisions. We have reviewed the existing trajectory-aware offloading decision algorithms for UAV-MEC systems. The main concepts, outstanding features, and important characteristics of each algorithm have been reviewed in a comprehensive manner. Notably, it has been found that RL-based algorithms have emerged prominently recently. From the comparison provided in the survey, trajectory-aware offloading schemes can now be better selected. Finally, we have discussed open issues and future research directions to expand the scope of this dynamic field. We believe that this survey will be beneficial for future research into and the development of trajectory-aware offloading frameworks in UAV-MEC systems.

## Figures and Tables

**Table 2:** Comparison of trajectory-aware offloading decision algorithms in terms of design principles.

Algorithm	Application Scenarios	Objective	Performance Metrics	Advantages	Limitations
DQN [15]	Emergency scenarios and hotspot areas	Minimize energy consumption and latency	Delay and energy	Simple and efficient	Can struggle with complex state spaces
DDQN [89]	Secure IoT tasks of remote area	Maximize the average secure computing capacity	Secure computing capacity	Reduces overestimation bias of DQN	Increased computational cost
DDPG [90]	Resource-limited end devices	Minimize the computational cost	Computation cost (energy)	Can handle continuous action spaces	Requires careful tuning of hyper-parameters
MADDPG [91]	Power IoT of remote area	Minimize the long-term average power consumption	Power	Can handle multi-agent environment	Difficult to train and can be unstable
MAPPO [92]	End devices with multi-type tasks	Minimize the weighted energy consumption	Energy	More stable than MADDPG	Can be slower to converge than MADDPG
MO-AVC [13]	Intelligent devices	Minimize latency and energy consumption of UAV, and maximize the quantity of collected tasks of UAV	Energy, delay, and number of collected tasks	Adaptive to uncertain environment	Hard to tune hyperparameters
GNN-A2C [93]	Remote end devices in complex environmental conditions	Maximize the computation tasks offloaded	Number of tasks	Can handle graph-structured state spaces	Computationally expensive
SCA [57]	Wireless power transfer-supported sensors	Reduce the total consumed energy by the UAV	Energy	Better performance for a large amount of data	High complexity
AO [86]	IoT devices with computation-intensive tasks	Reduce the total energy consumption of both UAV and UDs	Energy	Lower energy consumption	Very complex for multi-UAV
PDD [20]	Computation resource-limited mobile devices	Minimize total delay among all the users	Power, delay	High convergence rate for a moving UAV	Higher computational complexity
JSORT [87]	Mobile devices without computation infrastructures	Reduce the average weighted energy consumed by both UAV and SMDs	Energy	The system can deal with unpredictable environments	Does not consider the interference between data communication
BCD [88]	UAV-aided MEC support to resource-limited IoT devices	Maximize secure computing capacity	Computing capacity	Suitable for real-world decomposable problems	No guarantee for global convergence

**Table 3:** Comparison of trajectory-aware offloading decision algorithms in terms of operational characteristics.

Algorithm	Task Size	UAV Height (m)	UAV Speed (m/s)	Number of UAVs	User Mobility	Bandwidth (MHz)	Simulation Tool
DQN [15]	20–200 Mbits	–	–	1	Yes	10	PyTorch
DDQN [89]	–	100	–	2	–	1	Python 3.6 and Tensorflow 1.13.1
DDPG [90]	2–2.5 Mbits	–	–	1	Yes	1	Python 3.7.0 and Tensorflow 1.14.0
MADDPG [91]	$[16.8-25.2]\times 10^{4}$ bits	90	10	2–10	–	1	–
MAPPO [92]	3.5–4.5 Mbits	200	20	5	–	10	–
MO-AVC [13]	5 Mbits	80	–	5	Yes	–	PyTorch 1.6
GNN-A2C [93]	–	–	15	1	–	–	Python 3.9 and Tensorflow
SCA [57]	–	10	20	1	No	40	Matlab
AO [86]	400 Mbits	10	20	1	–	30	–
PDD [20]	10–50 Mbits	100	50	1	–	1	–
JSORT [87]	–	10	10	1	No	10	Matlab

Note: “–” means that the information is not mentioned in the corresponding publication.

## Data Availability

Data are contained within the article.
